# Association of matrix metalloprotease 1, 3, and 12 polymorphisms with rheumatic heart disease in a Chinese Han population

**DOI:** 10.1186/s12881-018-0538-4

**Published:** 2018-02-20

**Authors:** Wei Hu, Yujia Ye, Yirui Yin, Peng Sang, Linhua Li, Jing Wang, Wen Wan, Rui Li, Xiangfeng Bai, Yuehui Xie, Zhaohui Meng

**Affiliations:** 1 Laboratory of Molecular Cardiology, Department of Cardiology, The First Affiliated Hospital of Kunming Medical University, Kunming, 650032 China; 2 Yunnan Institute of Microbiology, Yunnan University, Kunming, 650091 China; 3 Department of Cardiovascular Surgery, The First Affiliated Hospital of Kunming Medical University, Kunming, 650032 China; 4 Department of Mathematics and Computer Science, Basic Medical College, Kunming Medical University, Kunming, 650500 China

**Keywords:** Allele frequency, Extracellular matrix, Susceptibility

## Abstract

**Background:**

Rheumatic heart disease (RHD) is an autoimmune disease triggered by acute rheumatic fever (ARF). Matrix metalloproteinases (*MMPs*) play an important role in the modulation of immune responses. The purpose of this study was to evaluate the association of *MMP1*, *3*, and *12* promoter polymorphisms with RHD in a Han population in Southern China since the 3 genes are localized on the same chromosome and have a combined effect.

**Methods:**

DNA samples were obtained from 90 adult patients with RHD and 90 control subjects. Polymorphisms in *MMP1* (rs1799750), *MMP3* (rs3025058), and *MMP12* (rs2276109) were genotyped by direct sequencing. Differences in genotype and allele frequencies of these polymorphisms were compared between the cases and the controls using Unconditional logistic regression models and Chi-squared test.

**Results:**

The 2G/2G genotype of rs1799750 in *MMP1* was associated with a significantly higher risk of RHD when compared with the 1G/1G genotype (OR = 3.227; 95% CI:1.118–9.31; *p* = 0.03). The frequency of allele 2G was higher in patients with RHD compared to the controls (69.4% vs. 58.9%; *p* = 0.048) No significant differences in genotype and allele frequencies of rs3025058 in *MMP3* and rs2276109 in *MMP12* were found between the patients with RHD and the controls (*p* > 0.05).

**Conclusions:**

Our results suggest that rs1799750 in *MMP1* might be a risk factor for RHD in a Han population in Southern China, and individuals carrying the 2G/2G genotype are likely more susceptible to RHD. In contrast, rs3025058 in *MMP3* and rs2276109 in *MMP12* might not contribute to the risk of developing RHD in this population. Further studies with larger samples and other ethnic populations are required to confirm these findings.

## Background

As a consequence of untreated group A streptococcal pharyngitis, as many as 3–6% of individuals may develop acute rheumatic fever (ARF) [1], and 42–60% of those with ARF will eventually develop rheumatic heart disease (RHD) [2, 3]. According to the Global Burden of Disease (GBD) Study, it is estimated that there were 34,232,795 cases of RHD and 345,000 deaths from RHD globally in 2010 [4]. In China, the prevalence of RHD is around 1.86 cases per 1000 adults between 27 and 71 years of age [5]. Approximately 30–45% of children and young adults with ARF develop RHD due to permanent valvular damage [6]. The basic pathological change is degradation and remodeling of the extracellular matrix (ECM) [7], which is characterized by the destruction of glycosaminoglycans, proteoglycans collagen, and elastin [8, 9].

Matrix metalloproteinases (*MMPs*), which are members of the multidomain zinc endopeptidases family, are not only capable of degrading many ECM components associated with valvular remodeling and calcification [10], but they also can modulate immune responses by processing cytokines and chemokines to change their activity [11]. McQuibban GA et al. [12] demonstrated that the N-terminus of monocyte chemoattractant protein 1 (MCP1), MCP2 and MCP4 was cleaved by *MMP1* and 3 to produce antagonist factors which dampen inflammatory processes. Further study has shown that *MMP3* has a dual role in biphasic modulation of inflammatory mediator activity by cleaving Interleukin 1β precursor into active form and degrading the biologically active cytokine [13]. RHD is an autoimmune disease that is triggered by ARF [14]. We can speculate that *MMPs* may be involved in the pathogenesis of RHD through an immune mechanism. However, there are few reports on the relationship between *MMPs* and RHD. In the present study, we will investigate the effect of *MMPs* polymorphisms on RHD.

Genetic polymorphisms in the promoter region of *MMPs* genes play an important role in the predisposition of patients to various diseases by altering transcriptional activity [15–18]. In the promoter of the *MMP1* gene, an insertion (2G)/deletion (1G) polymorphism was detected at position − 1607 (rs1799750). It has been demonstrated that the 2G promoter processes higher transcriptional activity than the 1G promoter by binding more Ets-1 transcription factor [16]. This *MMP1* promoter polymorphism has been reported to be associated with oligodendroglioma [19], coronary artery disease [20], osteoarthritis [21], and lumbar disc disease [22].

Another insertion (6A)/deletion (5A) polymorphism has been reported at position − 1612 (rs3025058) of the *MMP3* promoter. The 6A promoter has a reduced transcription level due to its higher affinity to the repressor binding site [17]. This *MMP3* promoter polymorphism has been associated with osteoarthritis [23], lung cancer [24], and myocardial infarction [25].

A single nucleotide polymorphism (SNP) in the *MMP12* promoter region has been reported to influence transcriptional activity [18]. This A to G substitution polymorphism is located at position − 82 (rs2276109) adjacent to the transcription factor activator protein-1 (AP-1). It has been suggested that this SNP may be a risk factor for rheumatoid arthritis [26], systemic sclerosis [27], ovarian carcinoma [28], and ischemic stroke [29].

Although *MMPs* polymorphisms have been associated with various diseases, the role of genetic polymorphisms in *MMPs* has not yet been evaluated in patients with RHD. In addition, *MMP1*, *3,* and *12* are known to be adjacently localized on chromosome 11q22.3 [15] and these 3 loci are considered to act in cooperation with each other [30]. In the present study, we evaluated the associations of 3 *MMPs* polymorphisms, rs1799750 in *MMP1*, rs3025058 in *MMP3*, and rs2276109 in *MMP12*, with RHD in a Han population in Southern China.

## Methods

### Patients and control subjects

We recruited 90 patients with RHD (46 males and 44 females) from the Department of Cardiovascular Surgery at the First Affiliated Hospital of Kunming Medical University. Diagnosis of RHD was based on modified Jones criteria as well as on transthoracic echocardiography (Phillips Agilent Sonos 5500, Amsterdam, Holland). Patients with one of the following cardiac manifestations were diagnosed with RHD: mitral stenosis including mitral valve area ≤ 2 cm^2^; the presence of leaflet thickening, commissural fusion, and alteration of the subvalvular apparatus; aortic regurgitation (with some degree of stenosis, aortic valve area ≤ 1.6 cm^2^); and tricuspid regurgitation. Carditis was found in 100% of the patients with RHD. No arthritis, subcutaneous nodules, chorea, or erythema marginatum was found in the patients. Patients with other heart complications and/or other inflammatory conditions were excluded from the study. We also recruited 90 age-, gender-, and ethnically-matched, unrelated healthy volunteers (41 males and 49 females) from the same hospital as the control subjects. This study was approved by the Institutional Research Ethics Committee of Kunming Medical University and followed the guidelines of the Declaration of Helsinki. All study participants were of self-reported Han ancestry and signed informed consent forms.

### Genotyping

Genomic DNA was extracted from peripheral blood using E.Z.N.A.® Blood DNA Mini Kits following the manufacturer’s instructions (Omega Bio-Tek, Inc., Norcross, GA, USA). Based on the sequences of *MMP1* (GenBank accession number: AY769434), *MMP3* (GenBank accession number: AF405705)*,* and *MMP12* (GenBank accession number: AY856072) that were available from GenBank and using the Primer5 software (Premier Biosoft International, Inc., USA), the appropriate primers were designed and synthesized for *MMP1* (rs1799750), *MMP3* (rs3025058), and *MMP12* (rs2276109) polymorphisms (TSINGKE Biological Technology, Beijing, China; Table 1). PCR amplifications were performed in a 30 μL volume containing 30 ng of genomic DNA, 2 μM of forward primer, 2 μM of reverse primer, 9 μL of ddH_2_O, 0.2 mM dNTP, and 15 μL of 2× Ex taq™ Buffer and 1 U Ex taq™ DNA polymerase (TSINGKE Biological Technology, Beijing, China). The cycling conditions used for PCR amplification were as follows: pre-denaturation at 95 °C for 5 min followed by 35 cycles of denaturation at 95 °C for 30 s, annealing at 55 °C for 30 s, extension at 72 °C for 45 s, and a final extension at 72 °C for 5 min. The products were subjected to gel electrophoresis and visualized by Gelview (Bioteke, Beijing, China). The PCR products were sequenced using an ABI Prism 377 automatic sequencer (PE Applied Biosystems, Foster City, CA, USA). Sequence data were analyzed using DNAMAN software (Lynnon LLC, San Ramon, CA, USA).

**Table 1:** PCR and sequencing primers for the polymorphisms

Gene (polymorphism)	Forward primer	Reverse primer	PCR product size (bp)
*MMP1* (rs1799750)	5’-AGTGGCAAGTGTTCTTTGGTCTC-3’	5’-GTTCCACATTAAATTGTCTTGGGT-3’	495
*MMP3* (rs3025058)	5’-TTATCTATCAGGCTTTCCTCTAAAC-3’	5’-CTGTGGCAATAAGATCCCTATGA-3’	571
*MMP12* (rs2276109)	5’-GGATAGGTGGACGTAGAGG-3’	5’-CTTGCCAATTTCATAACAG-3’	601

### Statistical analysis

Statistical analysis was performed with SPSS version 17.0 (SPSS Inc., Chicago, IL, USA). Student’s *t* test was used to compare the mean age between the cases and the controls. A chi-squared test was used to compare the sex distribution between the cases and the controls and to test for the deviation of genotype distribution from the Hardy-Weinberg equilibrium. Unconditional logistic regression models were applied to compare the differences in the allele and genotype frequencies of the polymorphisms between the cases and the controls, adjusting for age, gender. *P* < 0.05 was considered statistically significant.

## Results

### Demographic and clinical characteristics of patients with RHD and control subjects

The mean age of the patients with RHD and the controls were 51.0 (±5.4) and 50.9 (±4.9) years, respectively (*p* = 0.921). Of the RHD patients, 51.1% were male and 48.9% were female and in the control group, 45.6% were male and 54.4% were female (*p* = 0.55) (Table 2).

**Table 2:** Demographic and clinical characteristics of patients with RHD and the controls

	RHD patients (*n* = 90)	Controls (*n* = 90)	*p value*
Age (years)	51.0 ± 5.4	50.9 ± 4.9	0.921
Sex			0.55
Male	46 (51.1%)	41 (45.6%)	
Female	44 (48.9%)	49 (54.4%)	
Clinical characteristics			
Carditis	90 (100%)	0	
Arthritis	0	0	
Subcutaneous nodules	0	0	
Chorea	0	0	
Erythema marginatum	0	0	

Age is represented as mean ± SD

*RHD* rheumatic heart disease, *SD* standard deviation

### Genotype and allele distributions of *MMP1, 3,* and *12* polymorphisms in patients with RHD and control subjects

The genotype distributions of the *MMP1*, *3,* and *12* polymorphisms did not deviate from the Hardy–Weinberg equilibrium in both the cases and the controls (*p* > 0.05).

The overall genotype frequencies of rs1799750 in *MMP1* were not significantly different between the cases and the controls (*p* = 0.072; Table 3). Compared to genotype 1G/1G, genotype 2G/2G had a significantly higher frequency in the cases than in the controls (45.6% vs. 34.4%; *p* = 0.03; OR = 3.227, 95% CI:1.118–9.31), No significant difference in genotype 1G/2G frequency was found between the cases and the controls (47.8% vs. 48.9%; *p* = 0.09; Table 3). Compared to allele 1G, the frequency of allele 2G was significantly higher in the cases than in the controls (69.4% vs. 58.9%; *p* = 0.048; OR = 0.644, 95% CI: 0.416–0.996).

**Table 3:** Genotype and allele distributions of *MMP1*, *3,* and *12* polymorphisms

Gene (polymorphism)	RHD n (%)	Controls n (%)	*p value*	OR (95% CI)
*MMP1* (rs1799750)				
Genotype			0.072^*^	
1G/1G	6 (6.7)	15 (16.7)	–	(Ref.)
1G/2G	43 (47.8)	44 (48.9)	0.09	2.455 (0.87–6.926)
**2G/2G**	**41 (45.6)**	**31 (34.4)**	**0.03**	**3.227 (1.118–9.31)**
Allele				
1G	55 (30.6)	74 (41.1)	–	(Ref.)
**2G**	**125 (69.4)**	**106 (58.9)**	**0.048**	**0.644 (0.416–0.996)**
*MMP3* (rs3025058)				
Genotype			0.509^*^	
6A/6A	65 (72.2)	67 (74.4)	–	(Ref.)
5A/6A	21 (23.3)	22 (24.4)	0.983	0.993 (0.498–1.981)
5A/5A	4 (4.4)	1 (1.1)	0.230	3.908 (0.421–36.242)
Allele				
6A	151 (83.9)	156 (86.7)	–	(Ref.)
5A	29 (16.1)	24 (13.3)	0.473	1.24 (0.689–2.231)
*MMP12* (rs2276109)				
Genotype			0.767^*^	
A/A	85 (94.4)	83 (92.2)	–	(Ref.)
A/G	5 (5.6)	7 (7.8)	0.569	0.708 (0.215–2.324)
G/G	0 (0.0)	0 (0.0)	–	–
Allele				
A	175 (97.2)	173 (96.1)	–	(Ref.)
G	5 (2.8)	7 (3.9)	0.576	0.717 (0.223–2.307)

The genotype and allele with *p* < 0.05 are shown in bold fonts

*OR* odds ratio, *CI* confidence interval, and *Ref*. reference

^*^p value calculated from 2 × 3 contingency table

No significant difference in the genotype and allele frequencies of rs3025058 in *MMP3* was found between the cases and the controls (*p* = 0.509 and 0.473, respectively; Table 3). Similarly, there was no significant difference in the genotype and allele frequencies of rs2276109 in *MMP12* between the cases and the controls (*p* = 0.767 and 0.576, respectively; Table 3). Notably, no genotype G/G was observed for rs2276109 of *MMP12* in both the cases and the controls.

## Discussion

Twin studies have found that the risk of ARF in monozygotic twins with a history of ARF is increased by more than 6 times compared to that of dizygotic twins [31]. These findings provide evidence for the involvement of a host of genetic factors in susceptibility to RHD, which is the sequel to ARF in endemic conditions. Genetic association studies have shown that methylenetetrahydrofolate reductase (*MTHFR*) C677T polymorphism is associated with RHD [32] and other studies have suggested genetic associations between promoter polymorphisms in angiotensin-converting enzyme (*ACE*) and interleukin 10 (*IL-10*) and RHD [33, 34]. In the present study, we evaluated the associations of 3 *MMPs* polymorphisms, *MMP1* (rs1799750), *MMP3* (rs3025058), and *MMP12* (rs2276109), with RHD in a Han population in Southern China. Our study is the first to suggest an association between *MMP1* (rs1799750) and RHD (Table 3). We showed that the *MMP1*–1607 2G/2G genotype was associated with a significantly higher risk of RHD when compared with the 1G/1G genotype (OR =3.227; 95% CI: 1.118–9.31; *p* = 0.03; Table 3), and the frequency of the 2G allele was higher in RHD compared to the controls (69.4% vs. 58.9%; *p* = 0.048; Table 3). Studies have shown that *MMP1* is expressed in all heart valves and that the mRNA transcript for *MMP1* is significantly increased in patients with chronic valvular disease [35, 36]. Further studies have indicated that *MMP1* and its inhibitors play an important role in the development of an abnormal ECM characteristic of RHD and the *MMP1*/TIMP-1 ratio correlates positively with the rheumatic mitral valve area [8, 37].

*MMP1* is located on chromosome 11q22 and produced by stromal fibroblast cells, macrophages, endothelial, and epithelial cells [38]. The level of *MMP1* expression can be influenced by an insertion/deletion of guanine at position − 1607 in the promoter region; a core-binding site (5′-GGA-3′) for the Ets family of transcription factors is created when 2 guanines are present instead of 1 guanine, leading to a higher expression of *MMP1* [16]. Our findings were in accordance with those of previous studies that suggested that patients who carry the 2G allele are predisposed to the development of several types of cancers, periodontitis, coronary artery disease, and peripheral arterial occlusive disease [39]. We hypothesized that the 2G allele in the *MMP1* polymorphism might potentially increase the level of protein expression and accelerate the degradation of ECM, which provides the molecular basis for valvular tissue remodeling and repair during the development of RHD.

This *MMP3* promoter polymorphism has been associated with osteoarthritis [22], lung cancer [24], and myocardial infarction [25]. The *MMP3* gene is located in the same chromosome region as *MMP1*. The *MMP3*–1612 polymorphism has been associated with serum *MMP3* titer in rheumatoid arthritis patients and congestive heart failure associated with RHD [40, 41]. Ye et al. first reported that the *MMP3*–1612 polymorphism was involved in the regulation of transcription by binding the transcriptional repressor protein [17, 42]. These findings suggest that this functional polymorphism could be involved in the pathogenesis of RHD; however, we did not find a significant association between the *MMP3*–1612 polymorphism and RHD in this study, possibly due to the limited sample size. In addition, we observed in the present study that the 5A homozygote was very rare (2.8%) in the Yunnan population (Southern China), which is consistent with the results from previous investigations in the Guangdong population (1.8%, Southern China) and the Hebei population (2.5%, Northern China) [43, 44]. However, in other studies from British, Iranian, and Lithuanian populations, the frequency of the 5A homozygote was 25.5, 35.5, and 26.2%, respectively [45–47]. These findings suggest an ethnic difference in the *MMP3* gene polymorphism.

The *MMP12* gene, *MMP1,* and *MMP3* are located near each other on the same chromosome. In addition, the *MMP12* expression has been reported to be upregulated in human heart valve disease [48]. Accordingly, we postulated that the *MMP12* polymorphism might play an important role in the pathogenesis process of RHD; however, the present study did not show a significant association between the *MMP12* polymorphism and RHD. The lack of a significant association of the *MMP12* polymorphism with RHD could indicate that the *MMP12* polymorphism might not be a major risk factor for RHD.

Although our study did not reveal correlation between *MMP3*–1612 6A/5A, *MMP12* -82A/G polymorphisms and RHD in Han population, studies have shown a combined effect of *MMP1*–1607 1G/2G, *MMP3*–1612 6A/5A and *MMP12*-82A/G polymorphisms associated with esophageal adenocarcinoma (EA) risk in Caucasian [30]. The cause of the different conclusions may be difference in study population, low sample size or poor control-patient matching. In addition, the expression level of other *MMPs* members were found elevated in patients with RHD, such as *MMP2* and *MMP9* [49, 50]. Elevated *MMP2* levels in patients with RHD may be involved in atrial remodeling and atrial fibrosis by modulating the balance between B-cell lymphoma 2 (Bcl-2) and Bcl-2-associated X protein (BAX). Increased *MMP-9* may facilitate cardiac remodeling in RHD by playing a compensatory role for the decreased insulin-like growth factor (IGF)-I levels. Thus other *MMPs* members should be included in the polymorphism study due to their different pathogenic mechanisms in RHD.

There were several limitations in the present study. First, this case-control study had a relatively small sample size that might limit the statistical power to detect potential real genetic associations even though the controls were matched to the cases for age and sex. Further studies with larger samples are necessary to confirm our results. Second, our study subjects were confined to the Han population in Southern China. Additional studies in other ethnic populations are warranted to confirm our findings. Third, we only assessed the association between single polymorphisms of *MMP1*, *3*, and *12* and RHD. Investigations into other polymorphisms in *MMP1*, *3*, and *12* or other *MMPs* genes are required to elucidate the roles of *MMPs* genes in the development of RHD. Fourth, we did not use different methods to validate our results, such as RT-PCR, ELISA and/or immunohistochemical analysis.

## Conclusions

In summary, we assessed the associations of 3 *MMPs* polymorphisms, *MMP1* (rs1799750), *MMP3* (rs3025058), and *MMP12* (rs2276109), with RHD in a Han population in Southern China. Our study is the first to suggest an association between *MMP1* (rs1799750) and RHD, but we did not find a significant association between *MMP3* (rs3025058) and *MMP12* (rs2276109) and RHD. Further studies with different research methods, larger samples and other ethnic populations are required to confirm our results.

## Abbreviations

ARF: Acute rheumatic fever
CI: Confidence interval
ECM: Extracellular matrix
GBD: Global Burden of Disease
MMPs: Matrix metalloproteinases
MTHFR: Methylenetetrahydrofolate reductase
OR: Odds ratio
RHD: Rheumatic heart disease
SD: Standard deviation
SNP: Single nucleotide polymorphism
